# Novel Avulaviruses in Penguins, Antarctica

**DOI:** 10.3201/eid2307.170054

**Published:** 2017-07

**Authors:** Víctor Neira, Rodrigo Tapia, Claudio Verdugo, Gonzalo Barriga, Sunil Mor, Terry Fei Fan Ng, Victoria García, José Del Río, Pedro Rodrigues, Cristóbal Briceño, Rafael A. Medina, Daniel González-Acuña

**Affiliations:** Universidad de Chile Facultad de Ciencias Veterinarias y Pecuarias, Santiago, Chile (V. Neira, R. Tapia, V. García, J. Del Río, C. Briceño);; Universidad Austral de Chile Facultad de Ciencias Veterinarias, Valdivia, Chile (C. Verdugo, P. Rodrigues);; Pontificia Universidad Católica de Chile, Santiago (G.P. Barriga, R.A. Medina);; University of Minnesota College of Veterinary Medicine, St. Paul, Minnesota, USA (S. Mor);; University of Georgia College of Veterinary Medicine, Athens, Georgia, USA (T.F.F. Ng);; Icahn School of Medicine at Mount Sinai, New York, New York, USA (R.A. Medina); Millennium Institute on Immunology and Immunotherapy, Santiago (R.A. Medina);; Universidad de Concepción, Chillán, Chile (D. González-Acuña)

**Keywords:** avian paramyxovirus, penguins, Antarctica, avulavirus, zoonoses, viruses

## Abstract

We identified 3 novel and distinct avulaviruses from Gentoo penguins sampled in Antarctica. We isolated these viruses and sequenced their complete genomes; serologic assays demonstrated that the viruses do not have cross-reactivity between them. Our findings suggest that these 3 new viruses represent members of 3 novel avulavirus species.

Avian paramyxovirus (APMV) belongs to the genus *Avulavirus*, family *Paramyxoviridae*. There are 13 recognized *Avulavirus* species, each with 1 member, called avian paramyxovirus 1–13 (APMV-1–APMV-13) (*1*). A putative APMV-14 also has been recently described but not yet formally recognized (*2*).

In the past decade, APMV-10 through APMV-14 have been reported because of the intensification of surveillance of avian influenza A viruses (*3**–**6*). Most of the avulaviruses have been detected in wild birds associated with mild or no clinical disease; only Newcastle disease virus (a strain of APMV-1), APMV-2, and APMV-3 might cause substantial disease in poultry (*7*). Previous studies have described the presence of APMV-1, APMV-3, APMV-7, APMV-8, and other as-yet uncharacterized avulaviruses in Antarctic penguins (*8*). As a part of avian influenza surveillance expeditions in Antarctica during 2014–2016, we identified 3 novel avulaviruses in Gentoo penguins.

Cloacal, fecal, and serum samples were collected from Gentoo penguins (*Pygoscelis papua*) and Adélie penguins (*P. adeliae*), at 7 Antarctic locations (Technical Appendix Figure 1) during 2014–2016. Diagnostic tests, virus isolation, and serologic assays confirmed the identity of these paramyxoviruses (Technical Appendix).

We successfully isolated virus from 12 cloacal samples from Gentoo penguins on Kopaitic Island; these viruses showed positive hemagglutination titers ranging from 4 to 128 hemagglutination units. From these 12 isolates, only 5 were further confirmed by reverse transcription PCR and Sanger sequencing (*9*), suggesting the presence of new avulaviruses. All PCR-positive isolates were pooled and submitted for next-generation sequencing by using MiSeq 250 paired cycle run (Illumina, San Diego, CA, USA) (*10*).

By using next-generation sequencing, we obtained the genomic sequences of 3 novel avulaviruses that were named as follows: Antarctic penguin virus A (APVA), Antarctic penguin virus B (APVB), and Antarctic penguin virus C (APVC) (GenBank accession nos. KY452442–KY452444). Genome lengths of the 3 new avulaviruses ranged from 14,926 to 15,071 nt. The 6 genes for avulaviruses (coding for the nucleoprotein, phosphoprotein, matrix protein, fusion protein, hemagglutinin-neuraminidase protein, and RNA-dependent RNA polymerase protein) were identified in these virus genomes (Technical Appendix Figure 2, panel A). The sequence assembly was validated by coverage mapping (Technical Appendix Figure 2, panel B). The genomes described here are coding-complete; future experiments are needed to sequence the absolute terminus of the nontranslating region.

The 3 avulaviruses reported in this study showed 57%–60% genome-wide nucleotide identities to all other avulaviruses, as well as 32%–50% protein identities in the hemagglutinin-neuraminidase protein gene and 31%–48% in the fusion protein gene (Technical Appendix Figure 2, panel C). These new avulaviruses have 64%–67% genome-wide identity among each other. Accordingly, phylogenetic analyses (whether conducted by using genomes or specific genes) revealed that the new viruses form a monophyletic cluster with APMV-1, APMV-9, APMV-12, and APMV-13 (Figure; Technical Appendix Figure 3). Recently, a cutoff of <60% identity of nucleotide distance on whole genome has been proposed to differentiate avulaviruses (*3*); however, APMV-12 and APMV-13 and these 3 newly discovered viruses have higher identity. Thus, we suggest that this criterion requires further validation. 

**Figure:**
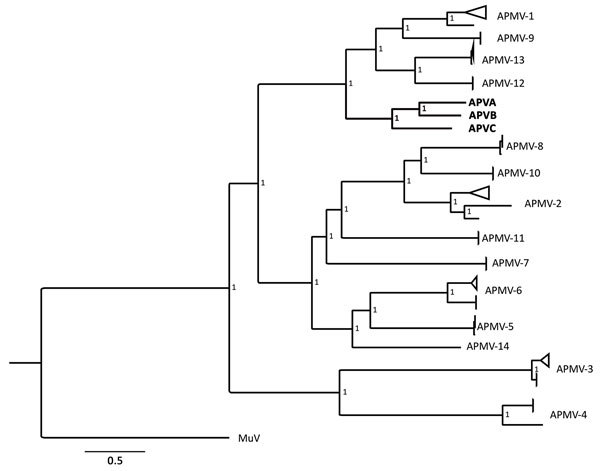
Bayesian phylogenetic tree based on concatenated nucleoprotein, phosphoprotein, matrix protein, fusion protein, hemagglutinin-neuraminidase protein, and RNA-dependent RNA polymerase protein gene sequences of 80 avulaviruses analyzed in a study of avulaviruses in penguins, Antarctica. Mumps virus was used as outgroup. Bold indicates the 3 novel viruses isolates in this study. The best-fit model of nucleotide substitution was generalized time reversible plus gamma plus invariant sites. The analysis was considered complete if the average SD of the split frequencies was <0.01 and effective sample size was >200. The values represent the posterior probabilities of each node. Scale bar indicates nucleotide substitutions per site. APMV, avian paramyxovirus; APVA, Antarctic penguin virus A; APVB, Antarctic penguin virus B; Antarctic penguin virus C; MuV, mumps virus.

Phylogenetic analysis and pairwise comparison suggests that APVA, APVB, and APVC might each represent novel avulavirus species, which we recommend naming *Avian avulavirus* *15*, *16*, and *17*, respectively (pending approval by the International Committee on Taxonomy of Viruses). We performed a hemagglutination inhibition assay by using APMV-1, APMV-2, APMV-3, APVA, and APVC antisera against isolates confirmed. No cross-reactivity was observed between APVA, APVB, and APVC. These viruses also did not show cross-reactivity against APMV-1, APMV2, and APMV-3 antisera. Antigenic results support the idea that novel viruses are 3 distinct species.

We observed cytopathic effects during infection of MDBK cells and Vero cells in all isolates evaluated. These effects were characterized by cell rounding and detachment of the monolayer, but syncytia were not evident (Technical Appendix Figure 4).

We also performed a hemagglutination inhibition assay by using APVA and APVC viruses. Three serum samples from Adélie penguins from Kopaitic Island reacted against APVC (titers 10–40), and 1 reacted against APVA (titer 40) (Technical Appendix Table). This result suggests that these novel avulaviruses can also infect Adélie penguins.

We report the successful virus isolation and whole-genome sequencing of avulaviruses in Antarctic penguin populations. Our analyses show that these viruses are genetically and antigenically divergent, indicating that Antarctic penguins harbor multiple avulaviruses. An important limitation is that the new viruses were not tested serologically against APMV-4 through APMV-13; however, genetic and antigenic differences between the new viruses support the idea that they are new species.

These data suggest that in Antarctica a much greater diversity of avulaviruses exists than previously recognized. Therefore, additional studies to evaluate the presence of these new viruses in other birds in Antarctica are needed to better understand the ecology and transmission of avulaviruses in this pristine environment.

Technical Appendix Methods and additional results in a study of avulaviruses in penguins in Antarctica.
