# Supplementary material for: Novel Avulaviruses in Penguins, Antarctica
Source: Emerg Infect Dis. 2017 Jul;23(7):1212–4. doi: 10.3201/eid2307.170054 (PMC5512496; doi:10.3201/eid2307.170054)
Supplement: Technical Appendix — Methods and additional results in a study of avulaviruses in penguins in Antarctica. [file 17-0054-Techapp-s1.pdf]

Article DOI: https://dx.doi.org/10.3201/eid2307.170054

Novel Avulaviruses in Penguins, Antarctica

Technical Appendix

Materials and Methods

Collection Samples

A total of 262 cloacal swabs, 83 fecal samples, and 138 blood samples were collected from two penguin species, Gentoo (*Pygoscelis papua*) and Adélie penguin (*P. adeliae*) (INACH RT 12–13), located in seven Antarctic stations during three scientific expeditions from 2014 to 2016. Blood samples were centrifuged at 800 g by 10 min and obtained sera were used for the hemagglutination inhibition assay (HIA) using isolated viruses from the present study. All samples tested negative to Influenza A virus (FLUAV) and Newcastle disease virus (NDV) using Real-Time Quantitative RT-PCR protocol. All protocols were approved by the Animal Ethics Committees of University of Chile (Pro. No. 150430002 and No. 02–2016) and University of Concepcion (Protocol No. CBE-48–2013).

Laboratory Tests

Cloacal and fecal samples were inoculated in SPF embryonic chicken eggs, and propagated Vero and MDBK (Madin Darby Bovine Kidney) cells to evidence cytopathic effect. Hemagglutination assay (HA) was used to detect hemagglutinant agents.

Cross reactivity was evaluated by hemagglutination inhibition assay (HIA) using APA, APC, APM-1, APMV-2 and APM-3 antisera. Briefly, APVA and APVC viruses were propagated and inactivated vaccines were prepared. To generate the antisera 2 guinea pigs per virus were immunized. Sera were collected after day 28. APMV-1, APMV-2 and APMV-3 antisera were kindly provided by Dr. Goyal at University of Minnesota.

Specific HIA was done with Antarctica penguin virus A and C isolated and tested against the sera obtained from Adélie penguins from Kopaitic Island.

Phylogenetic Analysis

Sequence alignment was performed by using MAFFT (*1*). Nucleotide sequence from each protein were aligned and then concatenated for further genetic and phylogenetic analyses. Maximum likelihood and Bayesian phylogenetic trees were inferred from sequence alignments by using PhyML 3.0 (*2*) and MrBayes v3.2.5 (*3*), respectively. The best-fit model of nucleotide substitution was identified by jModelTest (*4*). The robustness of ash analyses was assessed by bootstrap resampling process of 1,000 replications and when the average standard deviation of the split frequencies form the Markov chain Monte Carlo analysis was <0.01.

References

1. Katoh K, Standley DM. MAFFT multiple sequence alignment software version 7: improvements in performance and usability. Mol Biol Evol. 2013;30:772–80. PubMed http://dx.doi.org/10.1093/molbev/mst010

2. Guindon S, Dufayard JF, Lefort V, Anisimova M, Hordijk W, Gascuel O. New algorithms and methods to estimate maximum-likelihood phylogenies: assessing the performance of PhyML 3.0. Syst Biol. 2010;59:307–21. PubMed http://dx.doi.org/10.1093/sysbio/syq010

3. Ronquist F, Teslenko M, van der Mark P, Ayres DL, Darling A, Höhna S, et al. MrBayes 3.2: efficient Bayesian phylogenetic inference and model choice across a large model space. Syst Biol. 2012;61:539–42. PubMed http://dx.doi.org/10.1093/sysbio/sys029

4. Darriba D, Taboada GL, Doallo R, Posada D. jModelTest 2: more models, new heuristics and parallel computing. Nat Methods. 2012;9:772. PubMed http://dx.doi.org/10.1038/nmeth.2109

Technical Appendix Table. Sample sites and occurrence of avulaviruses in Antarctic penguins

	Gentoo penguin samples				Adélie penguin samples		
Location	Cloacal swab	Environmental sample	HA positive	RNA detected	Sera	HI Virus A	HI Virus C
Ardley Island	–	–	–	–	28	–	–
Arctowski Base	–	–	–	–	29	–	–
Kopaitic Island	132	–	12	5	81	3	1
Dorian Bay	–	22	–	–	–	–	–
Pleneau Island	–	44	–	–	–	–	–
Brown Base	–	17	–	–	–	–	–
*G.G.V. Base	130	–	–	–	–	–	–
Total	262	83	12	5	138	3	1

*Gabriel Gonzalez Videla.

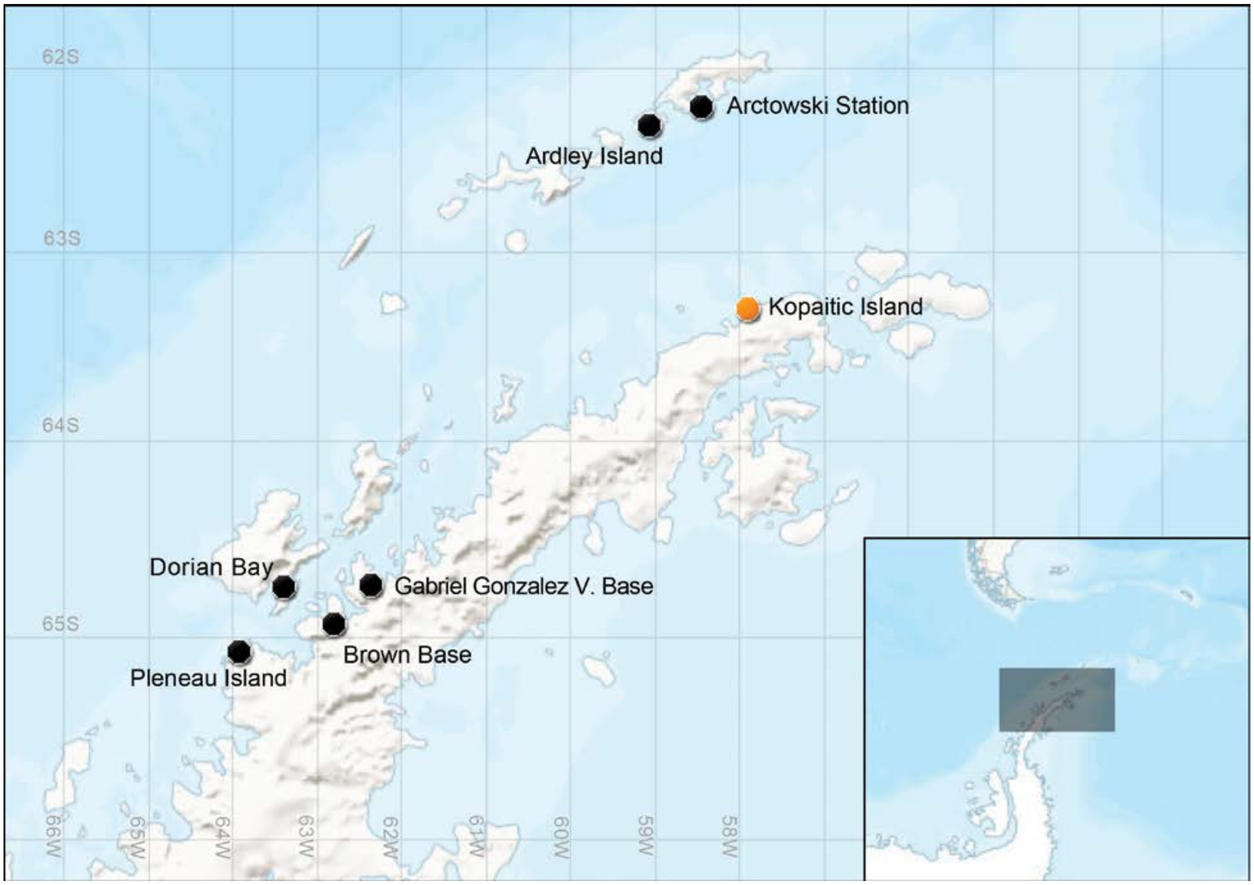

Technical Appendix Figure 1. Sampling locations on the Antarctica Peninsula. Orange dot indicate Kopaitic Island, location positive for Antarctic penguin avulaviruses.

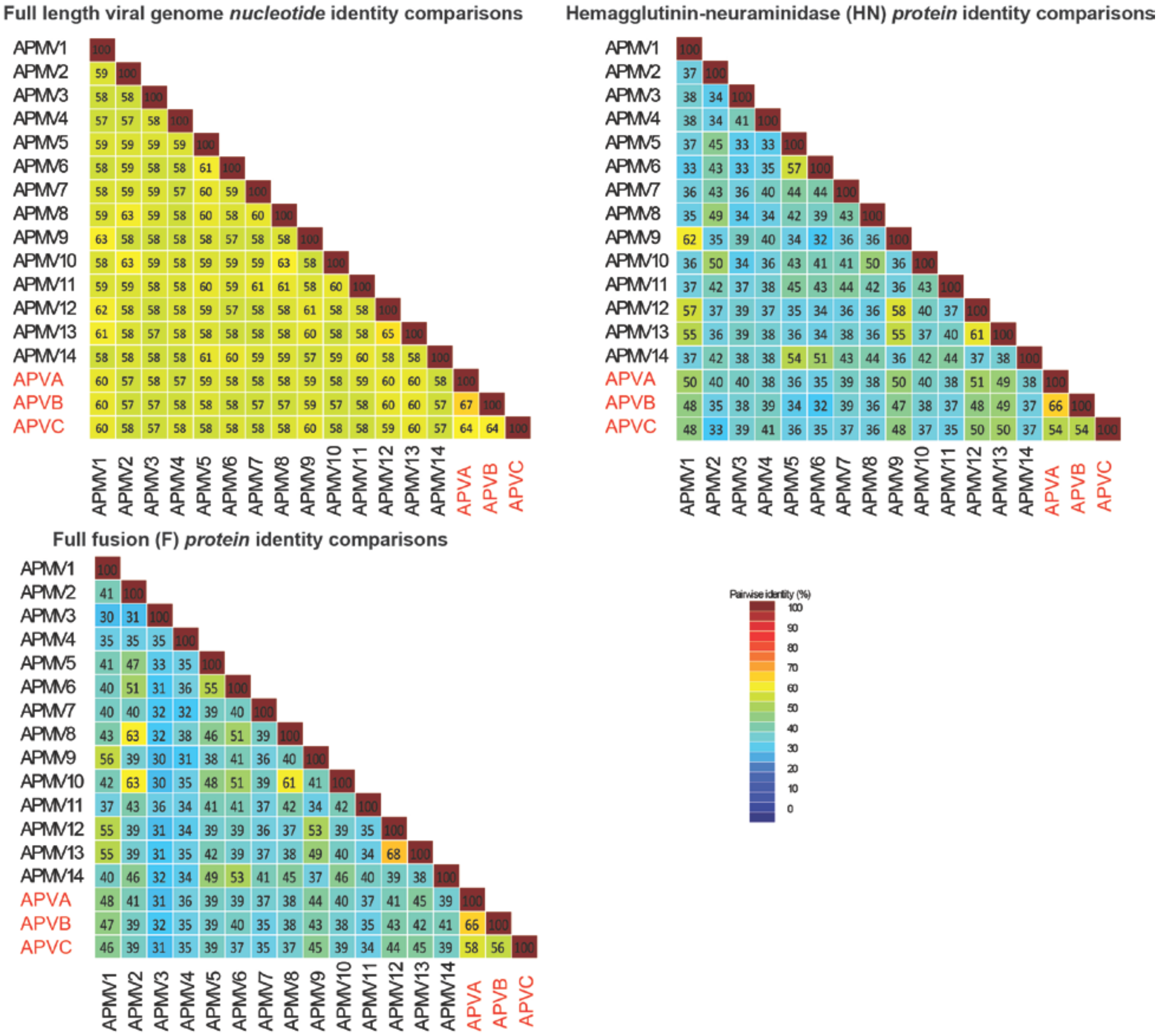

Technical Appendix Figure 2. Genetic characteristics of penguin avulaviruses. Gene acronyms were described in the main text. A) Genome organization of penguin avulaviruses, Newcastle disease virus is include as an example B) Sequence coverage of the three penguin avulavirus coding-complete genomes obtained by next generation sequencing, no misalignment was detected in mapping because the sequence identities between all three viruses were substantial C) Pairwise comparisons between the penguin avulaviruses and other avulaviruses. Each number represents the pairwise nucleotide or amino acid identities between the corresponding taxa.

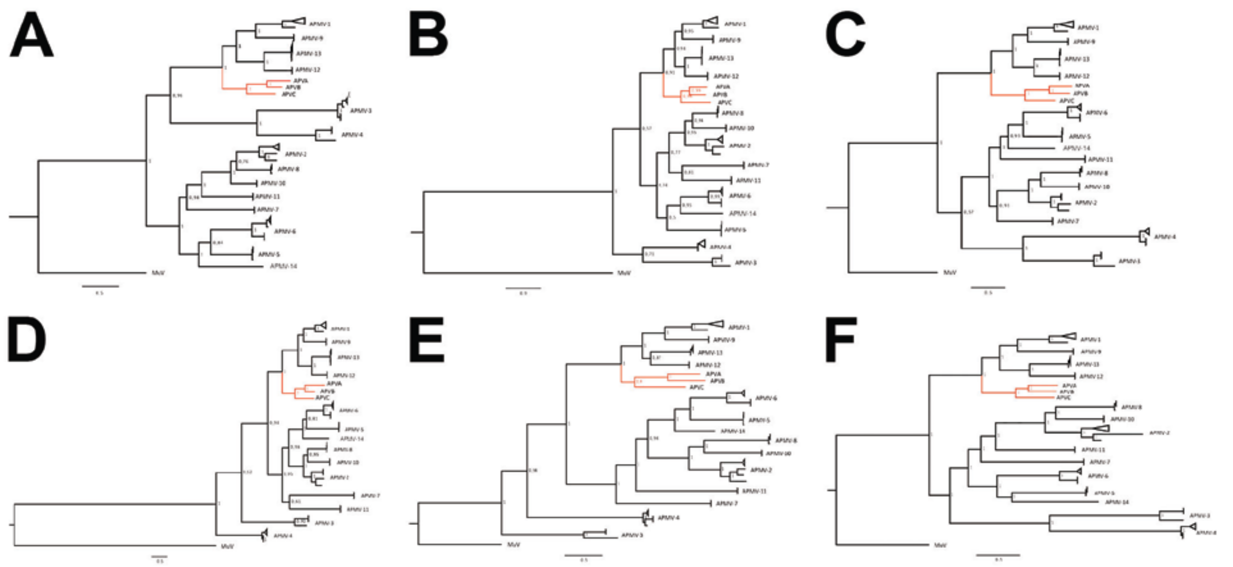

Technical Appendix Figure 3. Bayesian phylogenetic tree by gene using 80 avulaviruses reference sequences. The best-fit model of nucleotide substitution was GTR+I+G. The values represent the posterior probabilities of each node. Antarctic penguin viruses are colored in red. Mumps virus (MuV) was used as outgroup. A) Nucleoprotein gene B) Phosphoprotein gene C) Matrix gene D) Fusion protein gene E) Hemagglutinin-Neuraminidase gene and F) RNA-dependent RNA polymerase gene

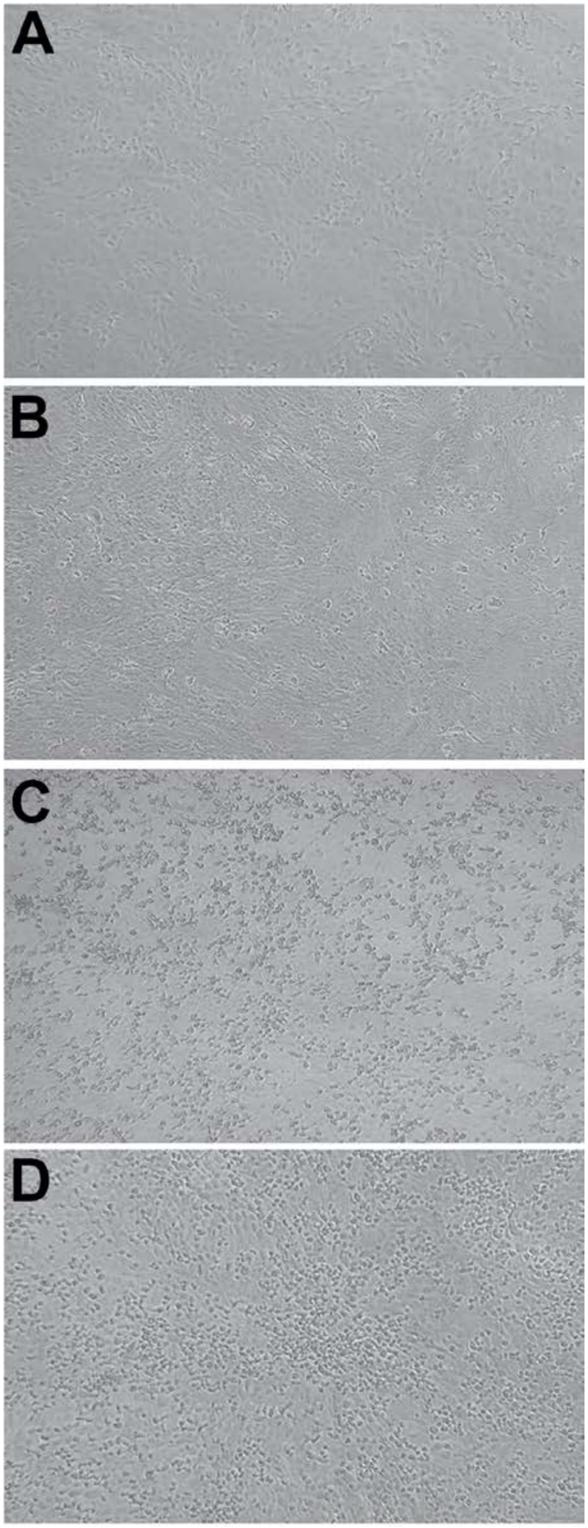

Technical Appendix Figure 4. Cytopathogenic effect of MDBK cells with Antarctic penguin virus A and C in absence of trypsin (200x) A) MDBK cells mock infected B) MDBK cells infected by Antarctic penguin virus A at 1 dpi C) MDBK cells infected by Antarctic penguin virus C at 1 dpi D) MDBK cells infected by Antarctic penguin virus C at 3 dpi.
